# Bacterial community analysis of anoxic/aeration (A/O) system in a combined process for gibberellin wastewater treatment

**DOI:** 10.1371/journal.pone.0186743

**Published:** 2017-10-20

**Authors:** Erming Ouyang, Yao Lu, Jiating Ouyang, Lele Wang, Xiaohui Wang

**Affiliations:** 1 School of Civil Engineering and Architecture, Nanchang University, Nanchang, China; 2 College of Chemical Engineering, Beijing University of Chemical Technology, Beijing, China; East China Normal University, CHINA

## Abstract

Gibberellin wastewater cannot be directly discharged without treatment due to its high concentrations of sulfate and organic compounds and strong acidity. Therefore, multi-stage anaerobic bioreactor + micro-aerobic+ anoxic/aeration (A/O) + biological contact oxidation combined processes are used to treat gibberellin wastewater. However, knowledge of the treatment effects of the A/O process and bacterial community structure in the aeration tank reactors of such systems is sparse. Therefore, this study was conducted to investigate the treatment effects and operation of the A/O process on gibberellin wastewater, as well as changes in the bacterial community structure of activated sludge in the aeration tank during treatment. Moreover, removal was examined based on evaluation of effluent after A/O treatment. Although influent chemical oxygen demand (COD), NH_3_-N and total phosphorus (TP) fluctuated, effluent COD, NH_3_-N and TP remained stable. Moreover, average COD, NH_3_-N and TP removal efficiency were 68.41%, 93.67% and 45.82%, respectively, during the A/O process. At the phylum level, *Proteobacteria* was the dominant phylum in all samples, followed by *Chloroflexi*, *Bacteroidetes* and *Actinobacteria*. *Proteobacteria* played an important role in the removal of organic matter. *Chloroflexi* was found to be responsible for the degradation of carbohydrates and *Bacteroidetes* also had been found to be responsible for the degradation of complex organic matters. *Actinobacteria* are able to degrade a variety of environmental chemicals. Additionally, *Anaerolineaceae_*uncultured was the major genus in samples collected on May 25, 2015, while *Novosphingobium* and *Nitrospira* were dominant in most samples. *Nitrosomonas* are regarded as the dominant ammonia-oxidizing bacteria, while *Nitrospira* are the main nitrite-oxidizing bacteria. Bacterial community structure varied considerably with time, and a partial Mantel test showed a highly significant positive correlation between bacterial community structure and DO. The bacterial community structure was also positively correlated with temperature and SO_4_^2-^.

## Introduction

**Fig 1:**
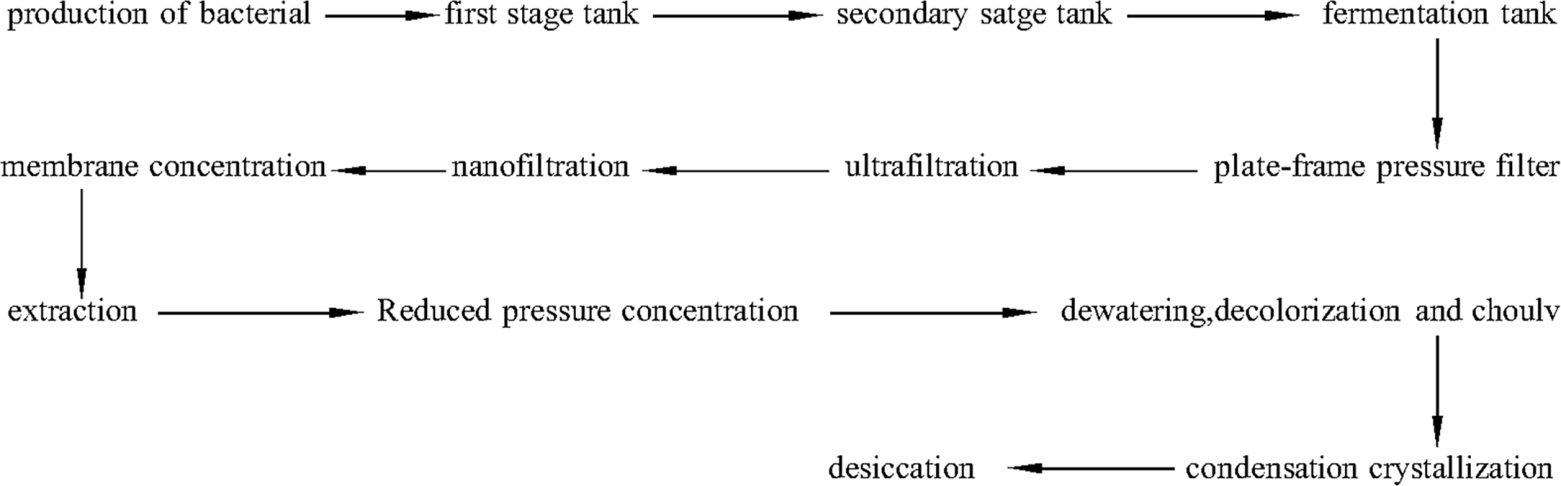
The production process of gibberellin.

Plant hormones are essential intrinsic regulators of plant development and growth [1]. Gibberellins not only promote internode elongation and cambial activity of stems [2], but also participate in the regulation of plant growth and development [3], including seed germination, stem elongation, flowering development, fruit growth, and root development. Gibberellin wastewater is generated by biological fermentation processes. The raw materials and methods for production of gibberellin result in gibberellin wastewater containing high concentrations of sulfate, organic compounds, and NH_3_-N, as well as strong acidity. These features make wastewater difficult to treat; therefore, a highly efficient treatment is required before discharge to prevent pollution. Fig 1 shows the production process of gibberellin.

High concentrations of sulfate have been found to have an inhibitory effect on microorganisms during biological treatment [4]. Nevertheless, Li. et al. [5] investigated the treatment of chemical synthesis-based pharmaceutical wastewater containing rich organic sulfur compounds and sulfate using an Upflow Anaerobic Sludge Blanket (UASB) reactor. They found that this method removed a considerable amount of COD and sulfate. The anaerobic/oxic (A/O) process is consists of a sequential anaerobic and aerobic stage for the biological phosphorus removal [6]. The A/O process has been widely used for sewage treatment in both urban and rural areas and is favored for its high efficiency and low energy consumption [7]. In this study, the concentrations of organic materials and sulfate were both high, and the composition of wastewater was very complex. The process for treatment of gibberellin production wastewater with a multi-stage anaerobic bioreactor combined with micro oxygen, A/O, and contact oxidation was established herein, while also applying chemical dosing to reduce the sulfate.

The specific purpose of this study was to investigate the impacts and operational conditions of the A/O process when applied for nutrient removal from gibberellin wastewater and to investigate changes in the bacterial community structure and diversity in activated sludge during treatment. The results demonstrated stability and effectiveness of the A/O process based on eight months of observation. Moreover, the bacterial community diversity in activated sludge was studied by Illumina MiSeq sequencing.

## Materials and methods

### Ethics statement

Permission for the samples was granted by the Jiangxi Ruifeng Biochemical Co. Ltd. (Jiangxi, China). The field studies also did not involve endangered or protected species.

### Raw water

The wastewater was obtained from a biochemical company located in Jiangxi province, China (115.478584°E, 27.900485°N). This company can produce 110 tons of gibberellin per year, accounting for more than 39% of the world's total gibberellin production. The gibberellin wastewater consists of three parts: raffinate wastewater, high concentration wastewater and comprehensive wastewater. In addition, the gibberellin wastewater contained high concentrations of sulfate and organic compounds, as well as strong acidity. The quality of gibberellin wastewater was shown in Table 1.

**Table 1:** Quality of gibberellin wastewater.

Wastewater		COD (mg/L)	SO_4_^2-^ (mg/L)	NH_3_-N (mg/L)	pH
**Raffinate wastewater**		40000	30000	170	2–3
**High concentration wastewater**		4800	1000	100	4–5
**Comprehensive wastewater**	**Acid and alkali production wastewater**	3000	100	——	5–6
	**Low concentration wastewater**	350	——	——	6.5–7.5
	**Domestic sewage**	250	——	35	7–7.5

### Start-up and operation of A/O system

**Fig 2:**
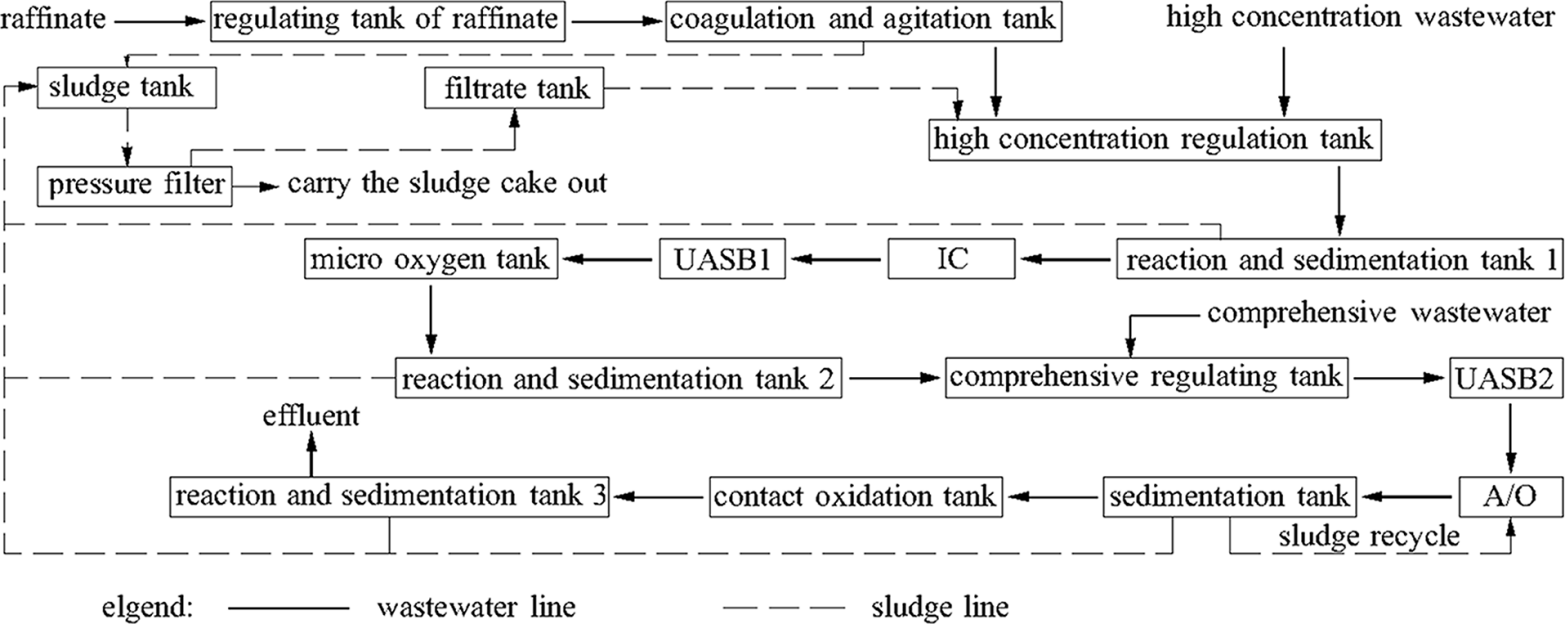
Process flow of wastewater treatment.

The A/O system could remove most of the NH_3_-N and a portion of the organics. The volume of A/O system was 2200m^3^ and the hydraulic retention time was 26.52h. The inoculum of in the A/O system is activated sludge and it was obtained from a previous company which produces gibberellin. The quantity of the sludge inoculation was 30 t. In the preliminary stage of the setup, the A/O system took in water once a day in the first day and the COD was kept at about 300 mg/L. The influent was stopped over the next two days and the A/O system stopped taking in water and kept on aeration. After the third day, the A/O system took in water twice a day. After operation for 15 days, the reactor was operated with continuous influent at half of the design flow. Additionally, the COD of the influent was strengthened during this time. After 25 days, there were large amounts of *Vorticella* sp. and rotifers in the reactor. The removal of COD and NH_3_-N was relatively high and the quality of the effluent was good. At this time, the influent COD was equal to the design loading of COD. After 30 days, the sludge became brown owing to flocculation, indicating successful reactor setup and good sludge activity. After setup, the reactor was used to treat the wastewater. During the setup period, the aerobic tank must remain aerated, and the dissolved oxygen should be maintained at 3 mg/L, while the pH was kept at about 6.5 to 8. Additionally, the pH of the anaerobic tank was maintained at 6 to 8 and the DO was less than 0.8 mg/L. Finally, KH_2_PO_4_ was added to the wastewater to maintain a COD:N:P ratio of 100:5:1. Fig 2 shows a schematic diagram of the treatment system.

### Microbial diversity analysis

#### Sample collection

A total of 12 samples were collected from the aeration tank. Samples were collected per half a month. Each sample was dispensed into a 1.5 mL sterile Eppendorf tube and centrifuged at 14,000×g for 10 min. The supernatant was decanted, and the pellets were stored at -20°C prior to analysis. For all samples, the sample numbers begin with the sample date.

#### DNA extraction and PCR amplification

Microbial DNA was extracted from samples using an E.Z.N.A.^®^ activated sludge DNA Kit (Omega Bio-tek, Norcross, GA, USA) according to the manufacturer’s protocols. The V4-V5 region of the bacterial 16S ribosomal RNA gene was amplified by PCR (95°C for 2 min, followed by 25 cycles at 95°C for 30 s, 55°C for 30 s, and 72°C for 30 s and then final extension at 72°C for 5 min) using primers 515F 5′-barcode- GTGCCAGCMGCCGCGG-3′ and 907R 5′-CCGTCAATTCMTTTRAGTTT-3′, which contained an eight-base sequence barcode unique to each sample. PCR reactions were performed in triplicate 20 μL mixtures containing 4 μL of 5 × FastPfu Buffer, 2 μL of 2.5 mM dNTPs, 0.8 μL of each primer (5 μM), 0.4 μL of FastPfu Polymerase, and 10 ng of template DNA.

#### Illumina MiSeq sequencing

Amplicons were extracted from 2% agarose gels and purified using the AxyPrep DNA Gel Extraction Kit (Axygen Biosciences, Union City, CA, USA) according to the manufacturer’s instructions, then quantified using QuantiFluor™-ST (Promega, Madison, WI, USA). Purified amplicons were pooled in equimolar amounts and paired-end sequenced (2 × 250) on an Illumina MiSeq platform according to the standard protocols. The raw reads were deposited into the NCBI Sequence Read Archive (SRA) database (Accession Number: SRP077353).

#### Processing of sequencing data

Raw fastq files were demultiplexed, then quality-filtered using QIIME (version 1.17) with the following criteria: (i) 300 bp reads were truncated at any site receiving an average quality score <20 over a 50 bp sliding window, discarding truncated reads that were shorter than 50 bp. (ii) Exact barcode matching, any sequences with two nucleotide mismatches upon primer matching or reads containing ambiguous characters were removed. (iii) Only sequences that overlapped by more than 10 bp were assembled according to their overlap sequence. Reads that could not be assembled were discarded.

Operational units (OTUs) were clustered with a 97% similarity cutoff using UPARSE (version 7.1 http://drive5.com/uparse/) and chimeric sequences were identified and removed using UCHIME. The taxonomy of each 16S rRNA gene sequence was analyzed by the RDP Classifier (http://rdp.cme.msu.edu/) against the silva (SSU115) 16S rRNA database using a confidence threshold of 70% [8].

### Statistical analysis

The Shannon–Wiener index was used to assess bacterial diversity. Principal component analysis (PCA) was conducted to examine variations among bacterial communities of these thirty-six samples. Canonical correspondence analysis (CCA) was used to illustrate relationship among operational conditions and bacterial community. Statistical analyses were performed using a VEGAN package in R (v.2.15.1; http://www.r-project.org/) and CCA was performed by Canoco for Windows 4.5.

## Results and discussion

### Effect of combined A/O process on nutrient removal from gibberellin wastewater

**Fig 3:**
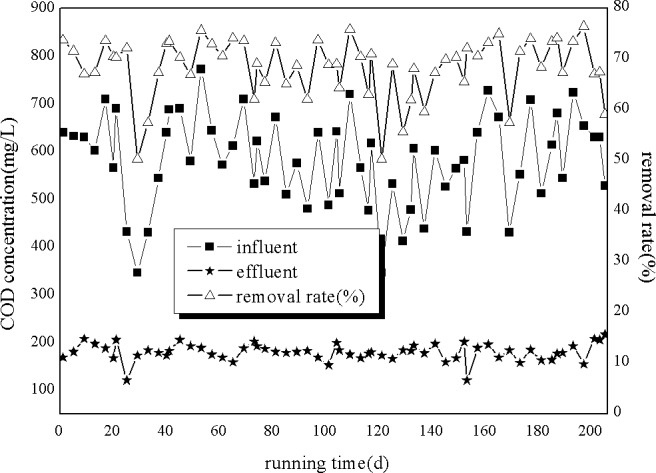
Removal of COD by A/O processes.

As shown in Fig 3, the influent COD concentrations fluctuated between 346 and 772 mg/L, with the lowest concentration (346 mg/L) being observed at 29 days and 121 days. Moreover, the highest influent COD concentration of 772 mg/L was observed at 53 days. The effluent COD concentrations fluctuated between 50.29 and 76.45. Taken together, these results indicate that removal efficiency changed with influent COD concentration. Moreover, the average COD removal efficiency was 68.41%.

**Fig 4:**
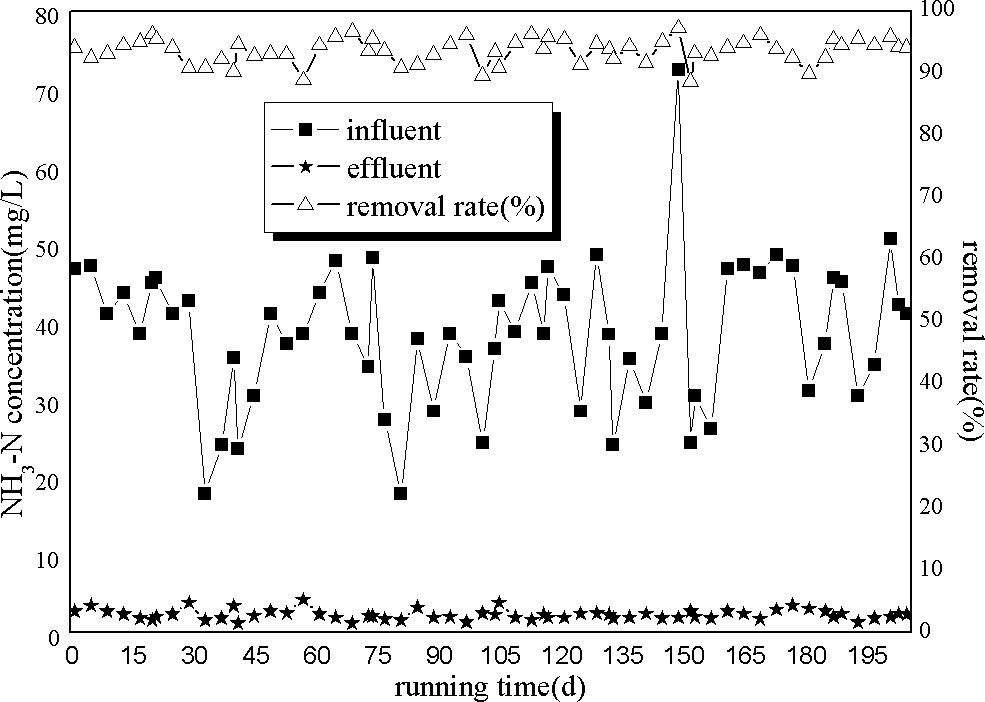
Removal of NH_3_-N by A/O processes.

As shown in Fig 4, the influent NH_3_-N concentrations fluctuated between 17.87 and 72.50 mg/L, with 17.87 being observed on days 33 and 81 and 72.50 being observed at 149 days. However, the effluent NH_3_-N concentration remained relatively stable, and the NH_3_-N removal efficiency was around 93.67%.

**Fig 5:**
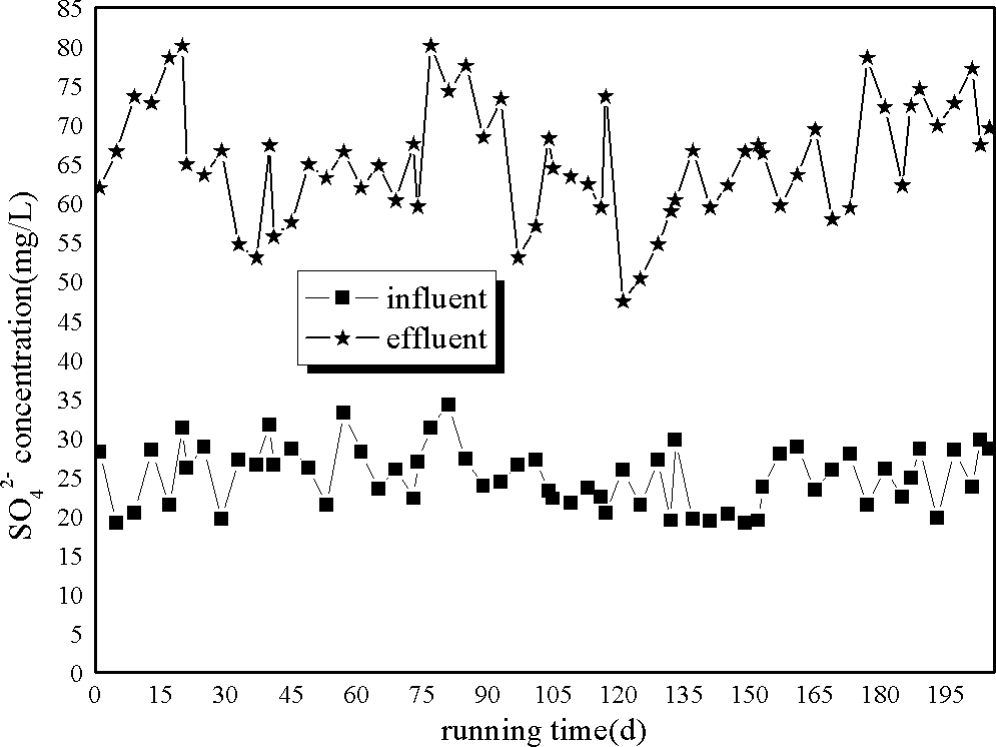
Removal of SO_4_^2-^ by A/O processes.

As shown in Fig 5, the concentration of effluent SO_4_^2-^ was higher than that of influent SO_4_^2-^, possibly because some of the SO_4_^2-^ had transformed into H_2_S, and some of the H_2_S had transformed into SO_4_^2-^ in the A/O system.

**Fig 6:**
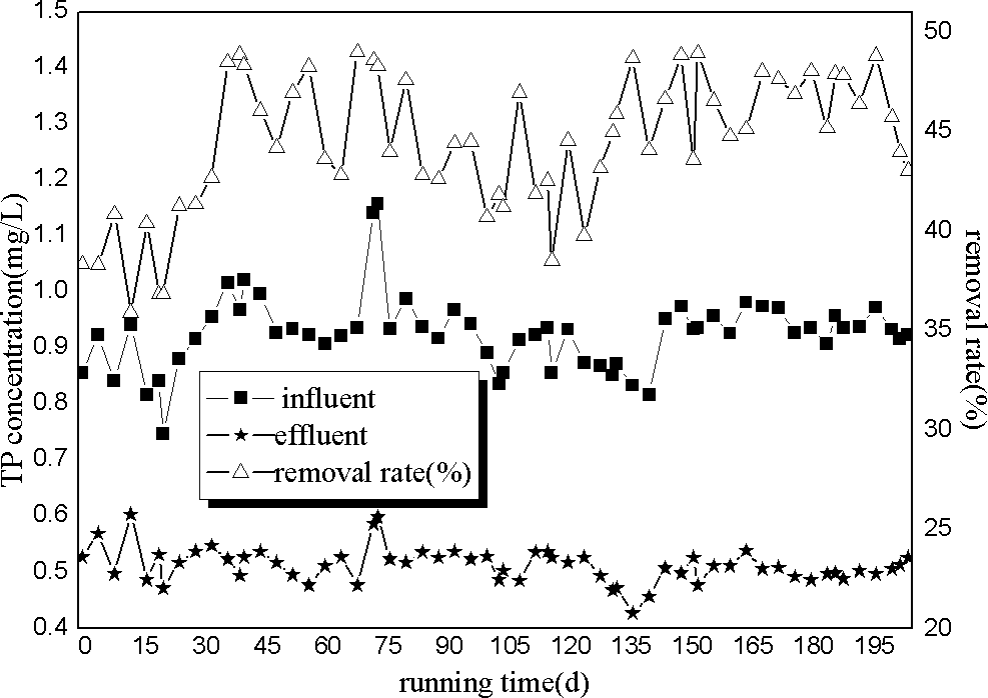
Removal of TP by A/O processes.

As shown in Fig 6, the influent TP concentrations fluctuated between 0.747 and 1.158 mg/L, while the effluent TP concentrations fluctuated between 0.428 and 0.604 mg/L. Moreover, the average TP removal efficiency was 45.82%.

These results show that A/O processes had good effects on gibberellin wastewater. Moreover, this method could remove most of the COD, NH_3_-N and TP with removal efficiencies of 68.41%, 93.67% and 45.82%, respectively.

### Illumina MiSeq sequencing results and microbial community structure

As shown in Table 2, there were 18,563 effective reads for 12 activated sludge samples. On average, the samples had 955.9 OTUs. The diversity of the bacterial community was determined based on the Shannon–Wiener index, which ranged from 4.81 to 5.43 (average = 5.18) [9]. These values are typical for diverse microbial populations without a few strongly dominant taxa [10].

**Table 2:** Diversity indices from 12 samples.

Samples	Sequences	OTUs	Shannon-Wiener
**20150525**	18563	824	4.81
**20150605**	18563	833	5.07
**20150625**	18563	813	4.93
**20150729**	18563	884	5.00
**20150813**	18563	1006	5.42
**20150828**	18563	954	5.28
**20150909**	18563	890	5.06
**20150925**	18563	968	5.31
**20151015**	18563	974	5.17
**20151117**	18563	1118	5.43
**20151120**	18563	1093	5.33
**20151205**	18563	1114	5.4

**Fig 7:**
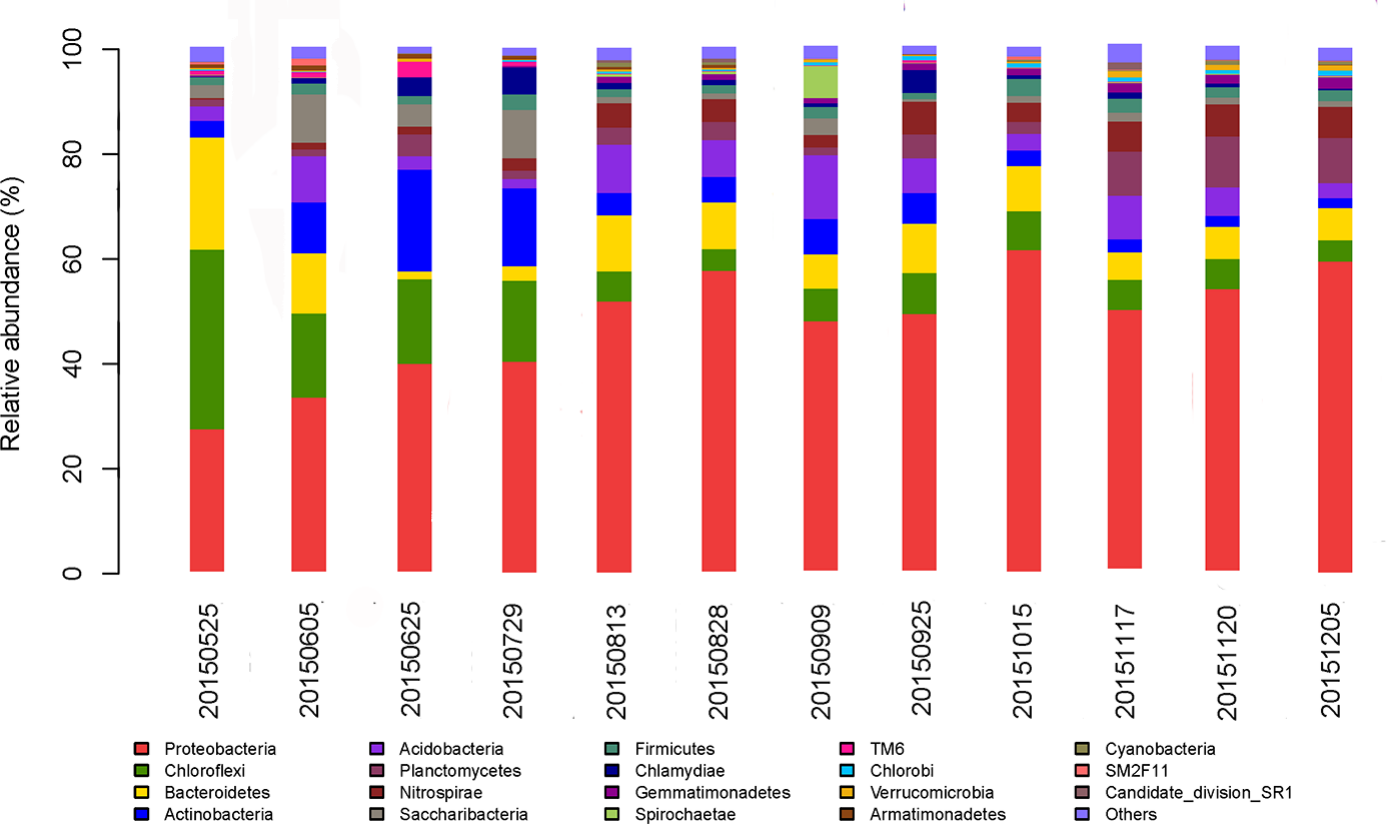
Abundances of different phyla in 12 activated sludge samples. The abundance is presented in terms of the percentage of total effective bacterial sequences in a sample.

As show in Fig 7, *Proteobacteria* was the dominant phylum in all samples, accounting for 27.19%–61.33% of the total effective bacterial sequences. These findings are concordant with those of our previous study, which showed that *Proteobacteria* was the dominant community member [11–15]. Evidence by Miura et al. [16] has shown that *Proteobacteria* was considered to be the most dominant phylum in MBR that played an important role in the removal of organic matter. The abundance of *Proteobacteria* obviously increased with operation of the aeration tank. Other dominant phyla included *Chloroflexi* (4.09%–34.25%), *Bacteroidetes* (1.56%–21.35%) and *Actinobacteria* (1.90%–19.37%). *Chloroflexi* has been detected as the predominant bacteria in anaerobic bioreactors and found to be responsible for the degradation of carbohydrates [17]. *Chloroflexi* also showed a great decrease with increased running time. Similar results were observed in previous studies that showed *Bacteroidetes* was the dominant community [18–21]. The anaerobes *Bacteroidetes* play an important role in the fermentation system to break down macromolecules such as protein, starch, cellulose and fiber [22]. *Bacteroidetes* also had been found to be responsible for the degradation of complex organic matters [23]. The relative abundance of *Bacteroidetes* decreased greatly at first, then increased to a relatively high abundance in sample collected on August 13, 2015, then finally stabilized. *Actinobacteria* play an important ecological role in recycling substances in natural ecosystems and are able to degrade a variety of environmental chemicals [24–26]. *Actinobacteria* was also the main phyla in a study conducted by Chen et al. [27]. The abundances of the dominant phyla were also different between samples. For example the abundance of *Chloroflexi* in sample collected on May 25 was significantly higher than that of other samples. *Planctomycetes* was the dominant route for ammonia removal in UASB reactor [28]. The relative abundance of anammox bacteria *Planctomycetes* increased with operation of the aeration tank and became one of the major phyla. Huang et al. [29] found that *Planctomycetes* was the major phylum in soil samples. Across different activated sludge samples, the relative abundances of some bacterial phyla, such as *Nitrospirae* and *Chlamydiae*, varied significantly. For instance, the relative abundance of the phylum *Nitrospirae* in sample collected on November 17, 2015 was significantly higher than in other samples. In contrast, the relative abundance of *Firmicutes* did not change significantly throughout the experimental period. Moreover, *Firmicutes* and *Gemmatimonadetes* have lower abundance in all activated sludge samples. This finding differed from those of previous studies, which showed *Firmicutes* and *Gemmatimonadetes* were the dominant phyla [30–32]. *Cyanobacteria* was a rare group in this study. Most *Cyanobacteria* can produce extracellular polymeric substances, mainly polysaccharide, which could adsorb heavy metals dispersed in the environment [33,34]. *Planctomycetes* and *Nitrospirae* increased greatly with operation time. *Proteobacteria*, *Chloroflexi*, *Bacteroidetes* and *Actinobacteria* are predominant phyla in the activated sludge.

**Fig 8:**
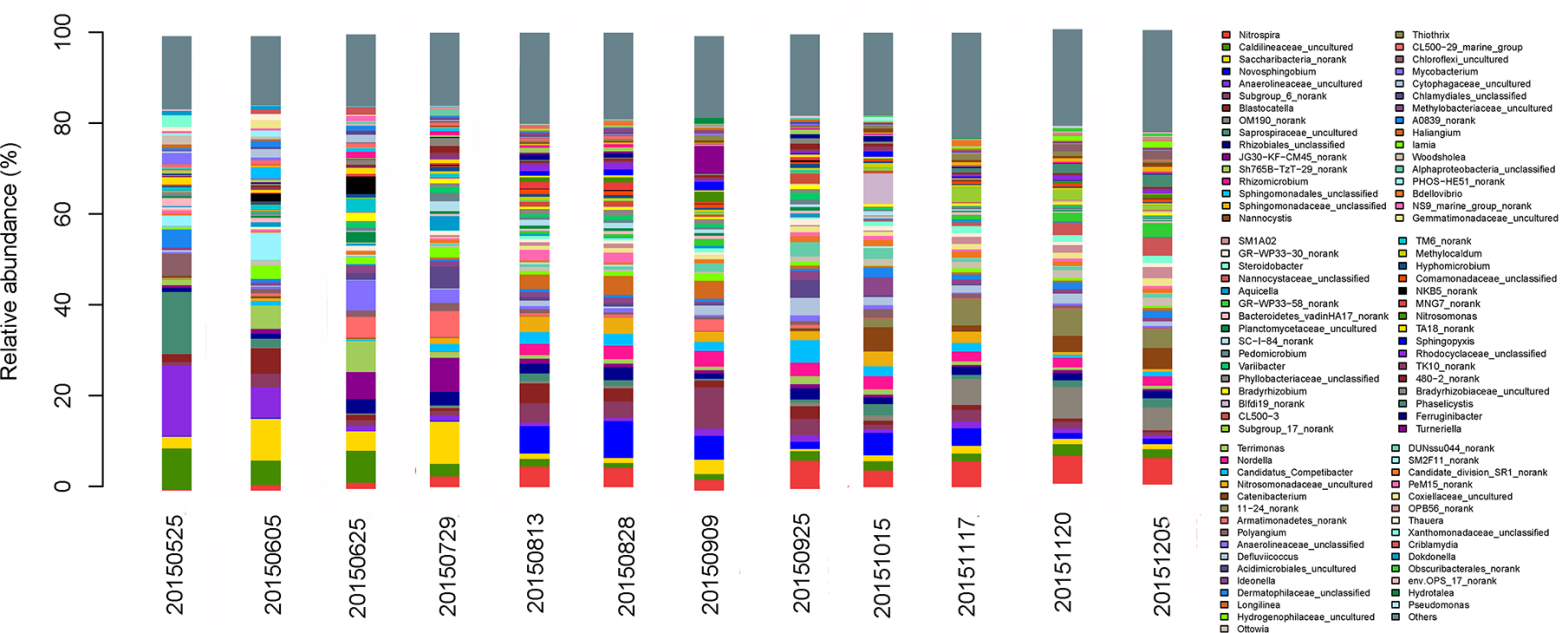
Taxonomic compositions of bacterial communities at the genus level in each sample retrieved from Illumina MiSeq pyrosequencing.

The distribution of sequences at the genus level in each sample is shown in Fig 8. The top 10 genera of each sample were selected, while all remaining genera were categorized as “other.” *Anaerolineaceae_*uncultured was the major genus in sample collected on May 25, 2015, while *Novosphingobium* and *Nitrospira* were dominant in most samples, especially during the latter portion of the experiment. These findings are consistent with those of previous studies that showed *Nitrospira* was the major phylum in the recirculating aquaculture system [35]. *Nitrosomonas* could be a minor component in activated sludge samples. *Nitrosomonas* are regarded as the dominant ammonia-oxidizing bacteria, while *Nitrospira* are the main nitrite-oxidizing bacteria in wastewater treatment plants [36]. The bacterial community composition showed little difference as the operating time increased in this study. *Anaerolineaceae_*uncultured and *Ottowia* decreased significantly as operating time increased. *Coxiellaceae_*uncultured was undetected in most samples. *Mycobacterium* was a minor component in most activated sludge samples, which was much different from the results of a previous study [37]. Some species of *Blastocatella* are aerobic, chemoorganotrophic organisms with a strictly respiratory type of metabolism [38]. The sulphur oxidizing bacteria genus *Thiothrix*, which increased significantly during the operation period, has also been found in aerobic granules incubated in brewery wastewater [39]. The relative abundance of *Caldilineaceae_*uncultured, *Saprospiraceae_*uncultured and *Anaerolineaceae_*uncultured decreased significantly as operation time increased, while *Nitrospira* increased greatly with increased operation time. *OM190_norank* did not change significantly in the first 8 samples, while it increased after October 15, 2015.

**Fig 9:**
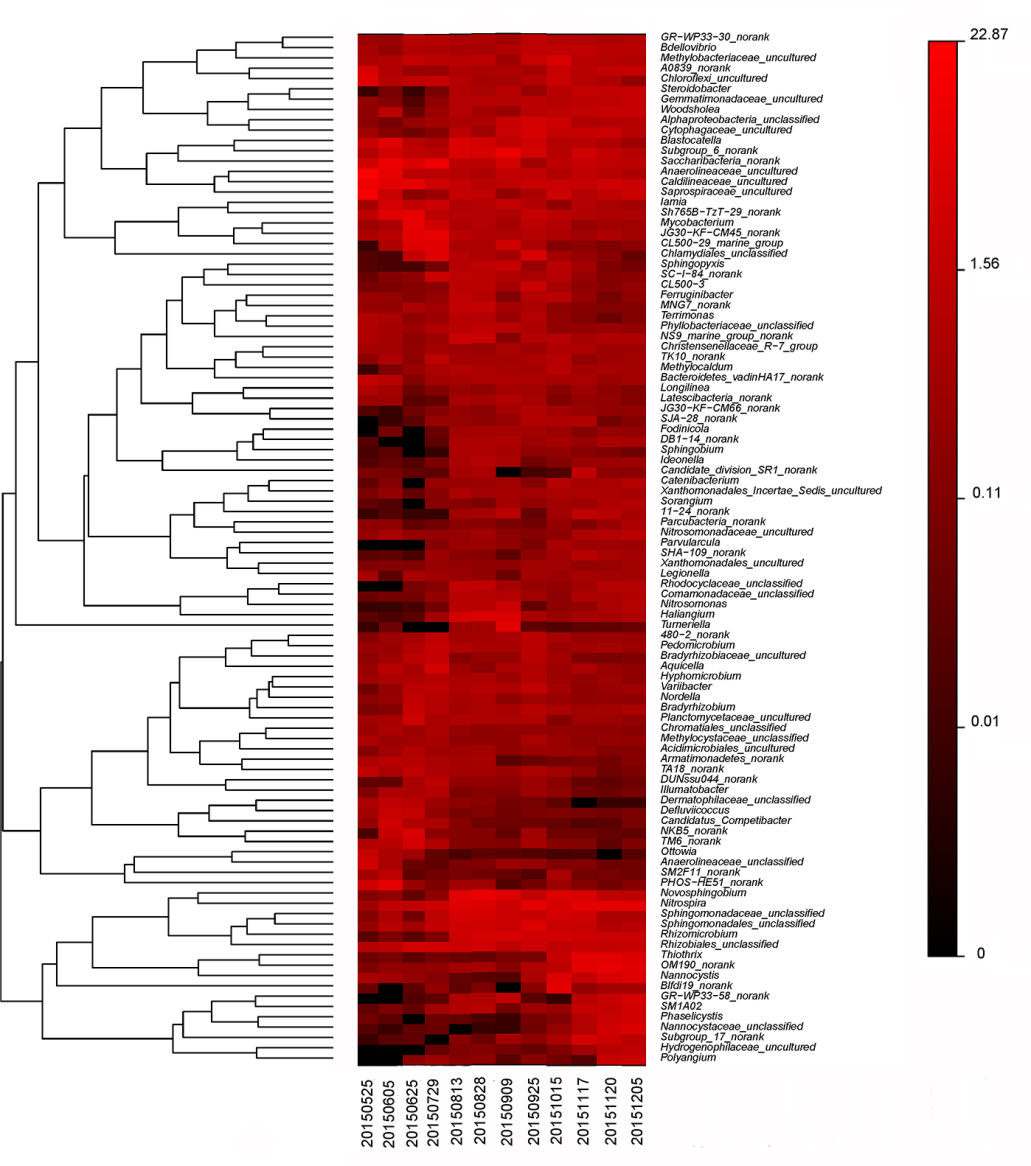
Heat map of top 10 genera in each sample. A total of 47 genera were selected from 12 samples. The color intensity in each panel shows the percentage of a genus in a sample based on the color key at the bottom of the figure.

The 10 most abundant genera in each sample were selected (a total of 47 genera for all 12 samples), and their abundances were compared with those in other samples (Fig 9). The bacterial population at the genus level differed significantly among samples from different time points. Most genera comprised a low proportion of the activated sludge samples. However, *Anaerolineaceae_unclassified* and *Anaerolineaceae_*uncultured were found in relatively high abundance in sample collected on May 25, 2015. Moreover, *Caldilineaceae_*uncultured had higher relative abundance in samples collected on May 25 and June 5, 2015. *Saprospiraceae_*uncultured had higher abundance in sample 20150525 than in other samples. *OM190_norank* was more abundant in samples collected on November 17 and 20 and December 12, 2015. In addition, *OM190_norank* clearly increased over the eight-month experimental period. *JG30-KF-CM45_norank* was abundant in samples collected on June 25 and July 29, 2015. *Nitrospira* had relatively high abundance in most samples. The relative abundance of *PHOS-HE51_norank* on June 5, 2015 was higher than that of other samples. *Saccharibacteria_norank* showed high relative abundance on June 5 and July 29, 2015.

**Fig 10:**
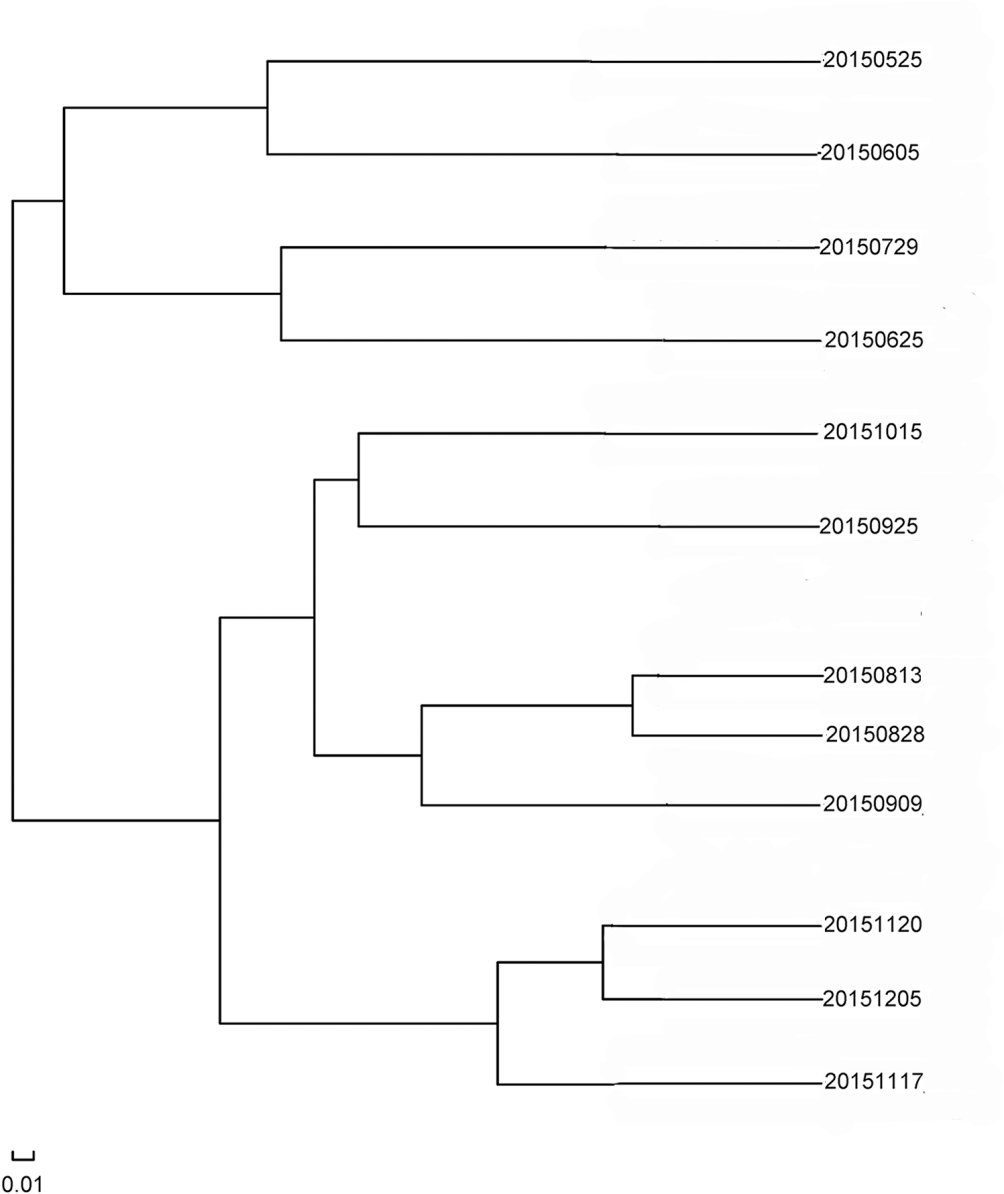
Multiple samples similarity tree.

Multiple samples similarity tree analysis was used to identify similarities and differences among the 12 bacterial community structures (Fig 10). Overall, samples collected from May, June and July formed one cluster, while those from August, September, October, November and December formed another cluster. The samples which were clustered together also indicated a more similar community structure between these samples. Additionally, the two clusters were well separated from each other, suggesting clear distinctions in the bacterial community structure of the two clusters.

**Fig 11:**
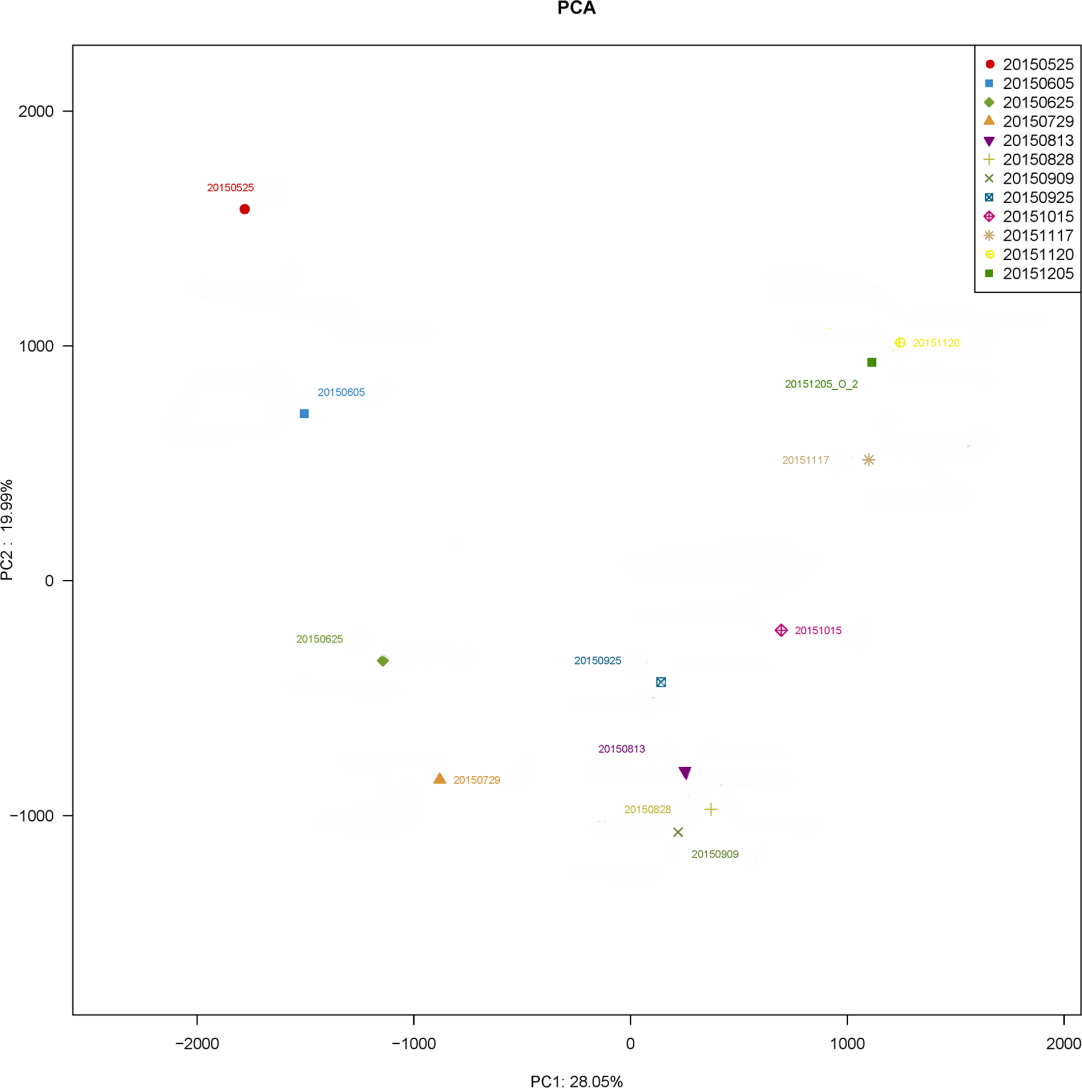
Principal component analysis (PCA) of bacterial communities from samples based on sequencing of Illumina MiSeq.

Principal component analysis (PCA) was conducted using high-throughput sequencing data to further corroborate the results of DNA fingerprinting [21] (Fig 11). The first axis explained 28.05% of the cumulative variance of species, while the second axis explained 19.99%. Samples collected on August 13 and 28, 2015 were closely related to sample collected on September 9, 2015, while sample collected on September 25 were closely related to sample collected on October 15 and those obtained on November 17, November 20 and December 15 clustered together.

**Fig 12:**
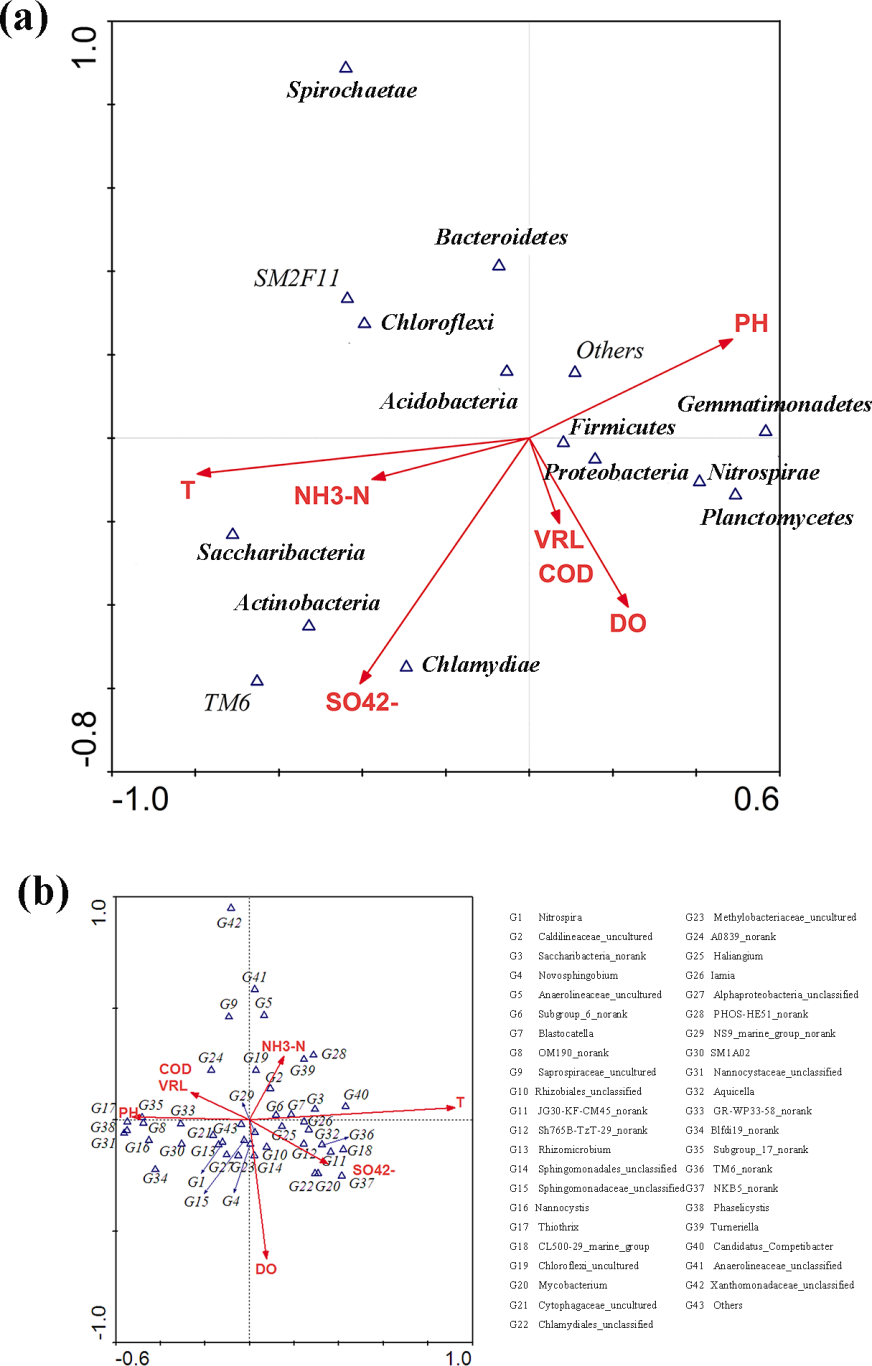
Correspondence canonical analysis (CCA) diagrams with bacterial community structure and operational conditions. (a) at phylum level (b) at genus level.

A canonical correspondence analysis (CCA) was used to illustrate relationship among operational conditions (S1 Table) and bacterial community (Fig 12). Based on the length of connecting wire, temperature, DO, pH and SO_4_^2-^ were found to be significantly correlated with the observed microbial composition both in phylum and genus level. In phylum level, *Proteobacteria* was positively correlated with DO and pH, and negatively with temperature. Phylum *Planctomycetes*, *Nitrospirae*. *Firmicute* and *Gemmatimonadetes* positively correlated with DO and pH, and SO_4_^2-^ and negatively with temperature. *Chloroflexi* positively correlated with temperature, and negatively with SO_4_^2-^,pH and DO. *Bacteroidetes* positively correlated with pH, and negatively with DO and SO_4_^2-^. *Actinobacteria* positively correlated with temperature and SO_4_^2-^, and negatively with pH. Besides, *Chlamydiae* positively correlated with temperature, SO_4_^2-^ and DO, and negatively with pH. As shown in Fig 12B, the abundance of *Nitrospira* (G1) positively correlated with pH and DO, and negatively with SO_4_^2-^ and temperature. The correlation of *Caldilineaceae*_uncultured (G2) was similar to *Saccharibacteria_norank* (G3) and *PHOS-HE51_norank* (G28),with positive correlation with temperature and SO_4_^2-^, and negative correlation with pH and DO. Abundance of *JG30-KF-CM45_norank* (G11) positively correlated with temperature,SO_4_^2-^ and DO, and negatively with pH, while abundance of *Thiothrix* (G17) had the opposite result. Genus *Novosphingobium* (G4) positively correlated with DO and SO_4_^2-^. Genus *Anaerolineaceae*_uncultured (G5) positively correlated with temperature, and negatively with SO_4_^2-^,pH and DO. The correlation of genus *OM190_norank* (G8) and *Saprospiraceae*_uncultured (G9) was similar, with positive correlation with pH, and negative correlation with temperature. Besides, *Saprospiraceae*_uncultured (G9) and *Anaerolineaceae_unclassified* (G41) also negatively with SO_4_^2-^ and DO.

## Conclusion

The combined processes of multi-stage anaerobic bioreactor + micro-aerobic + anoxic/aeration + biological contact oxidation were used to treat gibberellin wastewater. The A/O process led to good removal of COD, NH_3_-N and TP. *Proteobacteria* was the dominant phylum in all samples, followed by *Chloroflexi*, *Bacteroidetes* and *Actinobacteria*. *Novosphingobium* and *Nitrospira* were dominant genus in most samples. Illumina MiSeq sequencing showed that bacterial community structure and diversity changed with DO, temperature and SO_4_^2-^.

## Supporting information

S1 Table Operational conditions in 12 samples. (PDF)
