# Supplementary material for: Bacterial community analysis of anoxic/aeration (A/O) system in a combined process for gibberellin wastewater treatment
Source: PLoS One. 2017 Oct 20;12(10):e0186743. doi: 10.1371/journal.pone.0186743 (PMC5650175; doi:10.1371/journal.pone.0186743)
Supplement: S1 Table — (PDF) [file pone.0186743.s001.pdf]

S1 Table. Operational conditions in 12 samples

Sample	DO (mg/L)	T (℃)	COD (mg/L)	NH_3-N (mg/L)	pH	SO_4^{2-} (mg/L)	VRL(kg COD/(m^3 •d))
20150525	7.31	24	630	40.99	7.39	20.34	0.57
20150605	7.24	25.5	566	45.07	7.22	31.22	0.51
20150625	7.57	27.5	640	35.36	7.28	31.56	0.58
20150729	7.5	30.5	622	48.3	7.15	26.8	0.56
20150813	7.44	29.5	576	28.5	7.14	23.78	0.52
20150828	7.81	28	642	36.5	7.46	23.16	0.58
20150909	7.34	26	476	38.5	7.32	22.36	0.43
20150925	7.68	23.5	478	38.4	7.41	19.36	0.43
20151015	7.75	20	582	24.5	7.63	19.35	0.53
20151117	7.36	13.5	614	37.2	7.18	22.35	0.56
20151117	7.36	13.5	680	45.68	7.57	24.76	0.62
20151205	7.32	9	630	42.2	7.35	29.67	0.57
